# Considering and understanding developmental and deployment barriers for wearable technologies in neurosciences

**DOI:** 10.3389/fnins.2024.1379619

**Published:** 2024-03-13

**Authors:** Conor Wall, Yunus Celik, Victoria Hetherington, Peter McMeekin, Richard Walker, Lisa Graham, Rodrigo Vitorio, Alan Godfrey

**Affiliations:** ^1^Department of Computer and Information Sciences, Northumbria University, Newcastle upon Tyne, United Kingdom; ^2^Cumbria, Northumberland Tyne and Wear NHS Foundation Trust, Wolfson Research Centre, Campus for Ageing and Vitality, Newcastle upon Tyne, United Kingdom; ^3^Department of Nursing, Midwifery and Health, Northumbria University, Newcastle upon Tyne, United Kingdom; ^4^Northumbria Healthcare NHS Foundation Trust, Newcastle upon Tyne, United Kingdom; ^5^Department of Sport, Exercise and Rehabilitation, Northumbria University, Newcastle upon Tyne, United Kingdom

**Keywords:** wearables, validation, remote, internet of things, sustainability

## 1 Introduction

The advancement of wearable technologies (wearables) and their use in neurosciences signifies a new period filled with many possibilities and great potential (Byrom et al., 2018). Contemporary wearables incorporate a range of sensing modalities including but not limited to inertial measurement units (IMUs i.e., accelerometer and gyroscope), electroencephalogram (EEG), and functional near-infrared spectroscopy (fNIRS) which can provide high-resolution multimodal data to monitor significant (digital) clinical-based biomarkers or functional/performance outcomes pertaining to domains of interests within neuroscience. Notable examples include walking (i.e., gait performance) while simultaneously examining electrical brain activity or concentration changes of oxygenated and deoxygenated hemoglobin following neuronal activation (Stuart et al., 2018). Digital biomarkers or performance outcomes arising from wearables could aid a better understanding of neurological conditions like Parkinson's Disease (PD), epilepsy, or stroke during routine functional tasks (like walking) to get a better insight into real-world challenges and incidents arising from home and/or community-based activities. Moreover, integrating multi-modal wearable data (e.g., environmental) could enhance the understanding of real-world challenges for those with a neurological disorder (Johnson and Picard, 2020) e.g., unstable gait leading to near falls or falls during free-living walking (Warmerdam et al., 2020; Moore et al., 2023).

### 1.1 Going beyond the clinic

Traditionally, the evaluation of motor impairments in PD is often performed through visual observation by a clinician. However, that process is significantly dependent on the experience and subjective opinion of the clinician. To curb subjectivity, the use of specialized equipment, such as instrumented walkways has been adopted to obtain objective high-resolution data and greater insights. Yet, regardless of the effectiveness of the objective measures that process necessitates patients to travel to bespoke facilities, which can be inconvenient, costly, and physically challenging for those with mobility impairments (Syed et al., 2013). For a more decentralized approach, home motion systems (e.g., Microsoft Kinect) that include cameras, infrared, and radar-based devices have been utilized (Wang et al., 2014) but are costly and have limited widespread use (Demiris et al., 2009; Stone and Skubic, 2013). Accordingly, the need for low-cost/cost-effective technology became apparent. Achieving an economically decentralized approach would have added benefits of empowering patients to use these devices at home which in turn could ease the strain on healthcare systems to foster individualized care and independent living (Chen et al., 2023). Equally, the onus is no longer completely on the restrictive operational hours of clinics or the availability of medical professionals.

Wearables can provide a cost-effective and empowering approach for monitoring and rehabilitation beyond the clinic. They enable real-time assessment, potentially enabling healthcare providers to e.g., remotely monitor daily or weekly progress during rehabilitation exercises. Not only could wearables make rehabilitation more accessible by better accessing remote geographical areas, but they could also enable the personalization of treatment plans based on precise data tracking. However, the global push for technological integration and societal acceptance is an essential precondition for its widespread acceptance. Here, we broadly examine the technology-based challenges facing the use of wearables by care delivery teams or those working in clinical trials within neuroscience. It is worth noting that real-world deployment of wearables in care delivery and clinical trial settings face different regulatory pathways and the reader is directed elsewhere for those topics (Vasudevan et al., 2022; Izmailova et al., 2023; Leyens et al., 2024).

## 2 Clinical challenges

### 2.1 Validation

For wearables to be used consistently and reliably they must have a clearly defined contribution to improving clinical decisions while not dramatically increasing workload. For consistency and reliability, academic-based literature with robust evidence to show effectiveness in quantifying a physiological response is required. For example, quantifying gait in PD has emerged as a potential outcome to inform diagnosis and disease progression (Zhu et al., 2022). Validation of gait outcomes to known gold standard references is a fundamental must and/or investigation to determine any absolute differences due to any technology-based discrepancies between device comparison (Del Din et al., 2016). Recently, guidance on a flexible framework to inform clinical validation has been presented (Goldsack et al., 2020). In short, those creating, and using new wearables must follow proposed frameworks to show clinical effectiveness in their cohort of interest to convince associated healthcare professionals that adopting the technology has a real-world positive effect for their patients. Moreover, adoption of any wearable can only be truly achieved if it does not add extra burden to the healthcare professional to undertake patient assessments in what might be very time-limited meetings.

### 2.2 Perceptions and the future: education and upskilling

The successful trajectory of wearables within neurosciences is intertwined with perceptions and understanding (Karahanoğlu and Erbuğ, 2011). As with any technology, its widespread acceptance is contingent upon trust and comprehension of its functionalities. It's not solely about the technological proficiency of these devices but also about how their usability is determined by the public (Lee and Lee, 2020). For example, older adults often grapple with digital illiteracy and present a layer of complexity in this landscape (Wu et al., 2015). A well-informed public can discern the transformative capabilities of wearables, recognizing their potential to revolutionize healthcare, enhance daily life, and potentially unlock deeper health insights. Conversely, misconceptions or unwarranted fears, often stemming from a lack of understanding or misinformation, could hinder adoption (Cheung et al., 2019). Apprehensions may include privacy concerns, potential health implications, or even societal implications of widespread patient monitoring (Awotunde et al., 2021). To effectively engage older adults resistant to technology, a more personalized approach is required. For example, Xie (2011) proposed an initial assessment of technological skills to identify specific knowledge gaps and areas of discomfort. Following this assessment, training programs are tailored to the individual's needs and interests, focusing on practical technology applications they find engaging and relevant. To foster a conducive environment for the growth of wearable neurotech, proactive steps must be taken to educate where educational campaigns could play an important role. Leveraging various media platforms, from traditional outlets like television and newspapers to digital platforms such as social media and podcasts, could reach a diverse audience. Interactive workshops could offer hands-on experiences, allowing individuals to familiarize themselves with the technology (Yang et al., 2022). Furthermore, open dialogues in the form of talks at local community centers and societies or webinars can provide a platform for experts to address questions, dispel myths, and engage in constructive discussions about the future of wearables in healthcare (Shegog et al., 2020). However, public education is just one facet of the equation. As wearables become more integrated into healthcare and daily living, professionals interfacing with them may often require specialized training, so they can adequately inform patients how to use them in their homes (Bruno et al., 2018). Healthcare providers must be adept at interpreting arising data and understanding the application for altering patient care (Hilty et al., 2021). Caregivers could also need training, such as through video tutorials, to ensure optimal benefits for those in their care (Bruno et al., 2018; Li et al., 2019).

Upskilling should also extend to future healthcare professionals. Higher education institutions are beginning to employ the use of digital learning tools such as games, podcasts, e-compendiums, massive open online courses (MOOCs), and simulations. Those tools aim to produce better-skilled healthcare professionals, supplementing conventional teaching approaches with indispensable training in utilizing contemporary technologies that enhance patient care (Foss and Haraldseid, 2014). However, more adoption and integration into curricula is required to ensure the next generation of healthcare professionals is adept at dealing with a wearable revolution (Grimwood and Snell, 2020). It is essential that the future workforce is accustomed to the technology and what it is providing. That includes learning intricate functionalities and implications of wearables and how they can effectively utilize data (Brice and Almond, 2020). It is imperative they also have an awareness of how to integrate this information into personalized care plans, ensuring a balance between technological efficiency and human-centered care to ultimately improve patient care (Dyb et al., 2021). These unique challenges highlight the need for advanced but intuitive devices, where design prioritizes simplicity and intuitive interactions over mere technological feats (Noble and Blandford, 2013).

### 2.3 Data integrity: security, privacy, and user autonomy

The increasing connectivity of devices brings a concern: the secure handling of data. Wearables collect vast personal data, from activity patterns to sleep cycles. In malicious hands, those data could reveal an individual's habits and health, leading to threats like tailored phishing attacks (Peppet, 2014). Additionally, as wearables integrate with payment platforms and smart homes, breaches could expose financial data or allow unauthorized device control (Bezovski, 2016). Therefore, manufacturers have a responsibility to ensure comprehensive and evolutionary security measures (Safavi and Shukur, 2014). Central to this is the principle of clear data management, where users should be clearly informed about the handling of their data e.g., storage location, accessibility, and use (Troiano, 2016).

On a broader level, the healthcare industry faces its own set of challenges, highlighted by a report from the HIPAA Journal which noted 505 healthcare data breaches in 2019 alone, underscoring vulnerability to cyber-attacks (Alder, 2020). These breaches not only risk patients' personal information but also expose healthcare providers to legal actions from affected individuals seeking compensation for the invasion of privacy and potential harm. That emphasizes the necessity for robust data security measures, such as adopting advanced encryption, two-factor authentication, and conducting regular security audits. Those approaches should instill strong customer trust but may only be maintained through transparent communication and continuous technical support (Seh et al., 2020). However, the variability of data security and privacy regulations across jurisdictions poses a significant challenge for healthcare. Dimitropoulos and Rizk (2009) highlight the difficulty in accommodating varying policy requirements for health information exchange, stressing the need for a consensus on common policies to ensure adequate data protection and security.

Manufacturers should provide users with easily accessible and understandable privacy policies, ensuring that even those without technical expertise can grasp how their data are used (Gluck et al., 2016). Moreover, the principle of data minimization should be adopted. This means wearables should only collect data that is necessary for their functionality, thereby reducing potential harm if a breach occurs. For instance, if a fitness tracker's primary function is to measure steps, it shouldn't unnecessarily collect location data unless it's essential for a specific feature (Wolf et al., 2015). Finally, another crucial aspect is the implementation of user-controlled data permissions, where users should have the power to decide what data they are comfortable sharing and with whom. That empowers individuals but also fosters trust between users and manufacturers (Waldman, 2018).

### 2.4 Joined-up thinking: interoperability and IoT

The real value of this innovation emerges when wearables and peripheral technologies can communicate and work together. For example, heart failure events in patients with implanted devices were accurately predicted using a multi-modal approach with the integration of physical activity, heart rate, respiration rate, and other physiological variables (Boehmer et al., 2017). Another example could involve evaluating the relationship between sleep quality, heart rate variability, and stress levels during the day (Khanna and Jones, 2023). That scenario would require data fusion from various sensors, including actigraphy, respiratory sensors (e.g., nasal airflow sensors), galvanic skin response (GSR), body temperature, and heart rate sensors (Celik and Godfrey, 2023).

Currently, many proprietary systems exist, and each has its bespoke protocols and interfaces, resulting in compatibility issues. That fragmented environment poses challenges for users who wish to integrate wearables from different manufacturers in a seamless manner (Zeadally et al., 2019). Consequently, the need for standardization becomes essential, with Ravizza et al. (2019) providing a review of the current and upcoming regulatory requirements for wearable sensors in preclinical and clinical testing, highlighting the need for adherence to international standards set by bodies such as the International Electrotechnical Commission (IEC) and the International Organization for Standardization (ISO). Although some guidelines exist, e.g., Surface Electromyography for the Non-Invasive Assessment of Muscles (SENIAM) other physiological outcomes are lacking e.g., guidelines for inertial-based wearable placement (Celik et al., 2021). Key stakeholders should often prioritize collaboration over competition for a unified approach. By supporting standard protocols, wearables of any origin could communicate effectively, improving interoperability. Another potential approach to overcoming interoperability challenges involves standardizing the data outputs from various wearable technologies. This could be implemented similar to the model of the WHO's cytokine standards, which have facilitated uniform comparisons by setting a universal metric system (Mire-Sluis et al., 1998). That could also help facilitate, seamless integration and technological advancement by benchmarking against established standards to (i) understand data variations and normalize outputs and, (ii) enhance the precision and reliability of wearable healthcare technologies (Baker et al., 2017). Additionally, embracing open-source platforms could be a key driver for innovation beyond borders. Such collaborative efforts not only improve user experience but also enable a more inclusive and dynamic future for wearable neurotechnology (Bhat et al., 2019).

## 3 Developmental challenges

Some wearables can be expensive, making them elusive to a large proportion of society, where such financial constraints potentially deprive many of benefiting from the technologies ability to support a healthy life (He et al., 2023). For democratization of wearables, it's pivotal for manufacturers to innovate. That encompasses cost-effective production, scalable designs, and creating partnerships. Collaborations, especially with entities like governmental health bodies, can be instrumental in offsetting costs, ensuring wearables are universally accessible, and of benefit to the wider public (Kang and Exworthy, 2022). Manufacturers must also prioritize applications that offer the most substantial benefits and value. That concept, much like the evaluation of potential technology platforms in pharmaceutical pipeline management, would involve assessing wearables for their cost-effectiveness, patient outcome improvements, and efficiency in healthcare processes (Bode-Greuel and Greuel, 2005).

### 3.1 User-centric design: ergonomics, accessibility, and support

Continuing technological advancements can improve daily lives, but even advanced wearables can lose appeal if they don't align with user needs/preferences (Lidwell et al., 2010). That sentiment is accentuated in those with a neurological disorder, where people might be intricately navigating the challenges of physical or cognitive impediments (Wilson et al., 2022). Therefore, ergonomics is key. A device, irrespective of its capabilities, must be crafted to blend seamlessly into the user's daily routine. For instance, those with tremors might benefit from stabilization features or larger tactile buttons, while those with sensory sensitivities might prefer adjustable feedback settings or hypoallergenic materials (Imbesi and Scataglini, 2021; Kang and Exworthy, 2022). It is also essential that the wearables be comfortable for extended wear, ensuring they neither exert undue pressure nor evolve into a source of discomfort (Lewis and Neider, 2017). The user interface (tactile buttons, interactive touchscreens, or voice-activated commands), should be conceptualized with user ease at the forefront and should resonate as a harmonious extension of the user's intent, rather than an unwieldy appendage (Kumari et al., 2017). Furthermore, individuals grappling with functional limitations may experience difficulties in adeptly utilizing wearables that lack user-friendly fixation to their body, e.g., devices designed for lower back attachment. Consequently, these challenges serve to curtail their engagement with, and adoption of, such technological innovations (Guerra et al., 2023). Beyond the wearable itself, a robust support infrastructure can also play a pivotal role in amplifying the user experience. This includes available customer helplines, comprehensive online tutorials, and active community forums, where the primary goal is to ensure that users not only have access to a modern tool but are adequately supported in using it to its full potential (Vassli and Farshchian, 2018).

### 3.2 Evolution and sustainability: meeting the needs of tomorrow

In a time of rapid technological progress, there's a genuine concern about devices becoming outdated quickly (Spender et al., 2019). This poses a distinct challenge for manufacturers where they must now think ahead, creating devices that cater to current demands while also being prepared for future needs. This can be realized through regular software updates that bring new features and security, ensuring these devices remain current and reliable (Schukat et al., 2016). Another concern is battery life where a wearable's utility is significantly diminished if it cannot last a day without recharging (Godfrey et al., 2018). Manufacturers face the challenge of innovating while balancing power consumption with performance, especially since battery technology, despite its advancements, often lags the demands of modern devices, resulting in user frustrations. Solutions must encompass energy-efficient designs, the exploration of alternative power sources, and software optimizations (Rault et al., 2017). The emerging use of energy harvesting systems in wearable technology is spearheading this ethos, utilizing sophisticated methods to transform the body's kinetic energy, such as movement and gait, and thermal energy, like body heat differential, into electrical power to enhance the autonomy and longevity of wearables (Ali et al., 2023). For wearables to thrive, they must be forward-thinking and power-sustainable.

## 4 Conclusion

**Figure 1:**
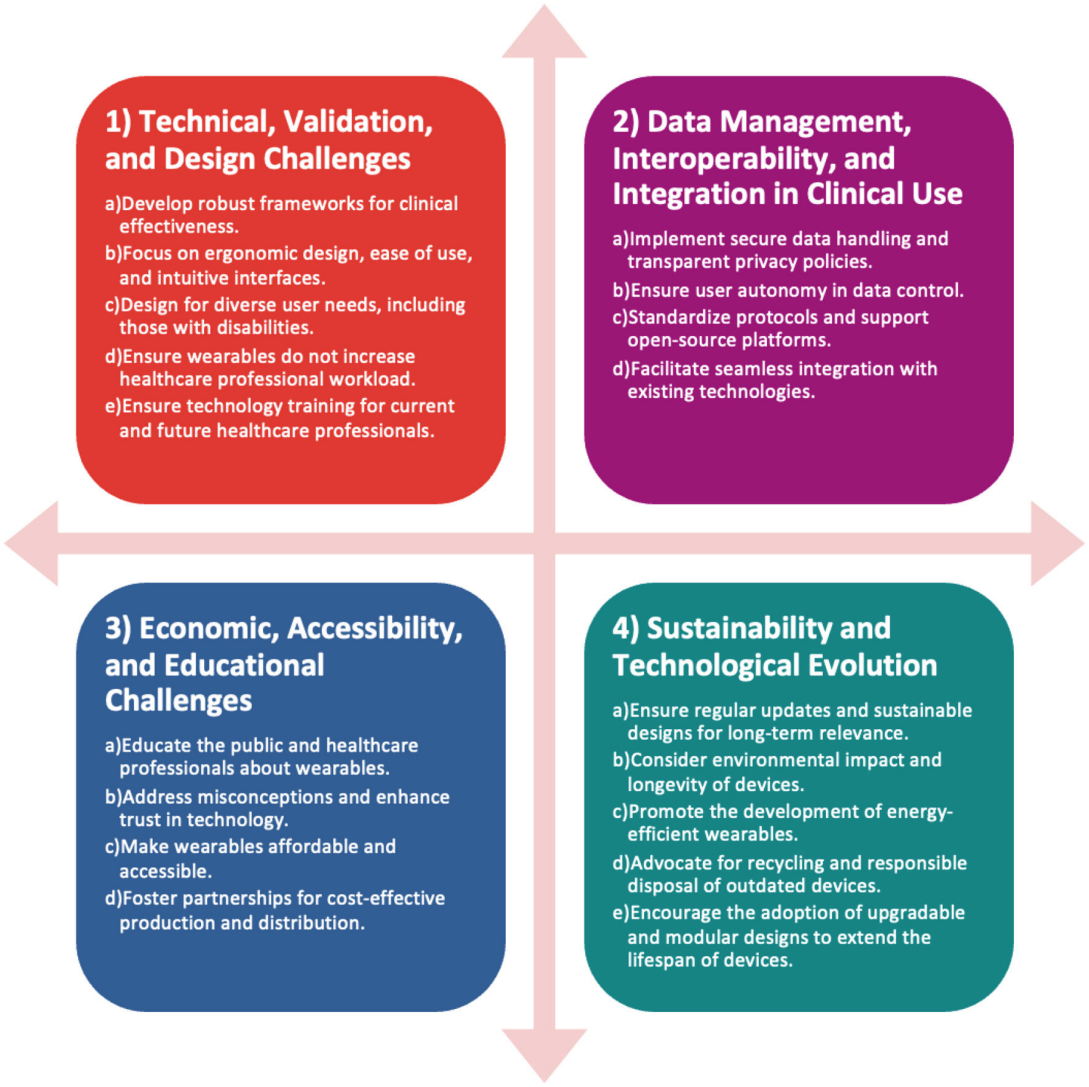
Key challenges and strategies for wearable healthcare technologies. This diagram classifies the challenges into four main categories: technical and design, data management, economic and accessibility, and sustainability, with a focus on ensuring effectiveness, secure integration, affordability, and long-term viability of wearables in healthcare.

The use of wearables has the potential to reshape the understanding of neurological conditions and revolutionizing patient care. Wearables, with their potential to monitor intricate biomarkers, could provide a plausible decentralized approach to healthcare. However, the journey to pragmatically integrate wearables into routine practice has clinical and developmental challenges: validation, perceptions, data integrity, lack of joined up thinking, weak user-centric designs, and evolutionary needs. Realizing wearable neurotech potential requires a concerted effort from manufacturers, healthcare professionals and the public (Figure 1). By addressing these challenges and fostering a collaborative environment, wearables can find their rightful place to unlock new possibilities in better understanding neurological conditions from beyond the clinic.

## Author contributions

CW: Writing – original draft, Writing – review & editing. YC: Writing – original draft, Writing – review & editing. VH: Writing – original draft, Writing – review & editing. PM: Writing – original draft, Writing – review & editing. RW: Writing – original draft, Writing – review & editing. LG: Writing – original draft, Writing – review & editing. RV: Writing – original draft, Writing – review & editing. AG: Writing – original draft, Writing – review & editing.
